# An improved overlap extension PCR for simultaneous multiple sites large fragments insertion, deletion and substitution

**DOI:** 10.1038/s41598-019-52122-8

**Published:** 2019-10-30

**Authors:** Wei Guo, Binhua Xie, Min Jiang, Xiao-Jing Zhu, Mengsheng Qiu, Zhong-Min Dai

**Affiliations:** 1 College of Life Sciences, Zhejiang University, 388 Yuhangtang Road, Hangzhou, Zhejiang 310058 P.R. China; 2 Institute of Life Sciences, Key Laboratory of Organ Development and Regeneration of Zhejiang Province, College of Life and Environmental Sciences, Hangzhou Normal University, 16 Xuelin Street, Hangzhou, Zhejiang 310036 P.R. China

**Keywords:** Synthetic biology, Molecular medicine

## Abstract

The existing molecular cloning methods are often limited by the availability of suitable restriction sites. It is still a challenge for simultaneous cloning of multiple fragments into different sites of a single vector. Here we developed a novel method named improved overlap extension PCR (IOEP) for restriction enzyme independent cloning of large fragments. The addition of primers enables IOEP to exponentially amplify the overlap extension product, thus greatly improves the amplification efficiency of large fragments. Moreover, coupled with the benefit of T4 DNA polymerase to improve cloning efficiency, our method can be used to simultaneously insert, delete and replace multiple DNA fragments at different sites.

## Introduction

The classical gene cloning requires the vector and the target fragment have compatible restriction endonuclease cleavage sites, thus the cloning site of a target gene is often limited^1,2^. Numerous restriction-free cloning techniques, including overlap extension PCR (OEP) methods^3–8^, *in vivo* recombination^9,10^ and exonuclease-based methods^11–18^, have been developed to overcome the site limitation of restriction endonuclease. However, due to the inefficient priming of megaprimer, OEP can be used only for inserts less than 6.7 kilobases (kb)^7^. Specific strains are required for *in vivo* recombination to enhance its efficiency^10^. The promising exonuclease-based methods utilize enzymes with exonuclease activity to generate single-stranded DNA ends for complementary annealing^11–18^, which mimic *in vivo* recombination. Here, we improved the OEP by supplying primers during OEP, which allows exponential amplification of OEP products. The exponentially amplified products can be treated with an enzyme having exonuclease activity such as T4 DNA polymerase^16,17^ for efficient cloning of large fragments. Our method can be further used for multiple insertion, deletion and replacement of large DNA fragments not only at a single site but also at multiple sites simultaneously.

## Results

### Improving overlap extension PCR by primed exponential amplification

**Figure 1:**
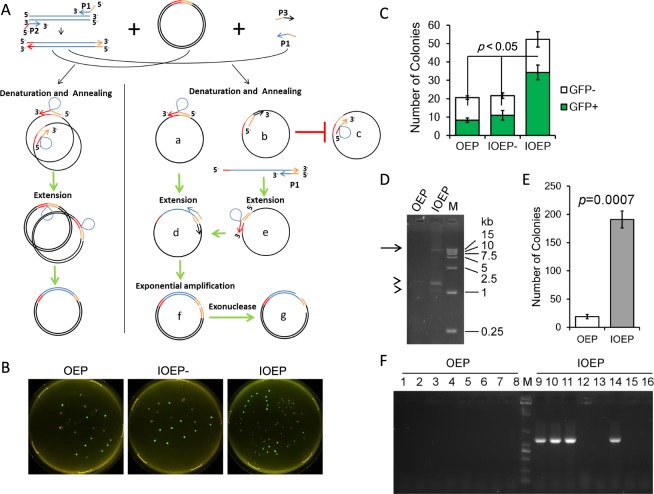
Improved overlap extension PCR (IOEP). (**A**) Features of IOEP. Left panel, traditional OEP uses chimeric primers to introduce overlap sequences into the PCR products, these PCR products were subsequently used as megaprimers for linear amplification. Right panel, additional primers were used in the IOEP. Three types of reaction occurred after denaturation. The type a and type c reactions are similar to that of traditional OEP. However, the type a reaction generates type d product, which can be used as template for exponential amplification in IOEP. The type b reaction is caused by the additional primers, which generates type e and subsequently type d product for exponential amplification. The type b reaction could also inhibit type c reaction. The amplified products could be treated with exonuclease to further increase its transformation efficiency. (**B**,**C**) Comparison the GFP incorporation efficiency by OEP, IOEP without T4 DNA polymerase treatment (IOEP-), and IOEP. Only OEP product was digested by DpnI to cut non-target plasmid. Treatment with T4 DNA polymerase greatly increased the number of GFP positive colonies. (**D**) Gel electrophoresis analysis showed that only IOEP was able to amplify a fusion fragment of about 12 kb. The target band was indicated by arrow, and the non-specific bands were indicated by arrowheads. The larger non-specific band is probably the un-incorporated insert. (**E**) Comparison of transformation efficiency. The number of clones obtained by IOEP is about 10 times that of OEP. (**F**) Half of the eight randomly picked colonies from IOEP contain correct construct, whereas none of the colonies from OEP is positive.

Traditional OEP utilizes chimeric primers to introduce overlapping sequences into the target fragment, then the amplified target fragment could be used as megaprimers for overlap extension (Fig. 1A). The inefficient incorporation of megaprimers results in linear amplification, thus limits the use of traditional OEP to fragments shorter than 6.7 kb (about 10 kb overlap extension product, consisting of 6.7 kb target and 3.3 kb vector^13^). We proposed an improved OEP (IOEP) by adding primers to the reaction, which could be used to exponentially amplify the overlap extension product, thus greatly improved the amplification efficiency of IOEP (Fig. 1A). Compared with OEP, only one additional primer is required, which contains about 20 bp complementary to vector at its 3′ end, and about 20 bp complementary to one of the OEP primer at its 5′ end. Following exonuclease such as T4 DNA polymerase treatment, which promotes complementary annealing of DNA ends, the cloning efficiency of IOEP could be further increased^17^.

We tested the efficiency of IOEP with and without (IOEP-) T4 DNA polymerase treatment and compared it with OEP by incorporation of GFP into pBlueScript II KS (−). Our results showed that a similar number of GFP positive and negative colonies were formed between OEP and IOEP- (Fig. 1B,C). The number of colonies, especially the GFP positive ones, was significantly increased following T4 DNA polymerase treatment (Fig. 1B,C). Although DpnI was not used in IOEP- and IOEP to deplete non-target plasmid, the number of GFP negative colonies remains at a low level as the exponential amplification of IOEP is highly efficient.

To further test the efficiency of the IOEP, we compared IOEP with OEP to obtain a 12 kb lentivirus vector, which is constructed by replacing a 241 basepairs (bp) fragment with 1336 bp copGFP-WPRE fragment in the LCas9 plasmid. There is difficulty in PCR amplification of the entire LCas9 plasmid, which contains long terminal repeats and a highly GC-rich region^19^. Due to the difficulty in amplification and size limitation, OEP couldn’t be used to amplify the 12 kb target. However, a clear 12 kb fragment was obtained by IOEP (Fig. 1B), indicating that our method can be used to efficiently amplify large and difficult –to-amplify overlap extension product. After DpnI and T4 DNA polymerase treatment respectively for the OEP and IOEP products and transformation, we found that the number of colonies obtained by IOEP was about ten times that of OEP (Fig. 1C). We randomly picked 8 colonies for PCR screening. As a result, none of the eight randomly selected colonies obtained from OEP contained the target gene fragment (Fig. 1D). Whereas even without DpnI treatment to reduce original plasmid, half of the colonies obtained from IOEP contained the target fragment (Fig. 1D), indicating that IOEP can be used efficiently for large vector construction.

### Simultaneous insertion, deletion and replacement of multiple fragments

**Figure 2:**
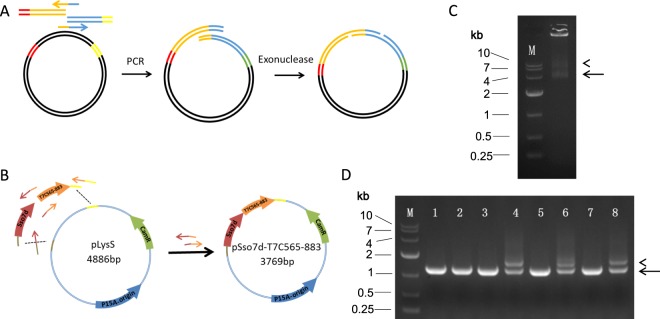
Use of IOEP for insertion of two fragments at one sites. (**A**) Scheme for substitution of one fragment by two fragments. (**B**) Diagram for construction of pSso7d-T7C565-883 plasmid. (**C**) PCR products of the pSso7d-T7C565-883 fusion. Specific band was indicated by arrow. The PCR may also resulted in concatemer as indicated by arrowhead. (**D)** Gel electrophoresis analysis showed that 5 out of 8 randomly picked colonies are positive constructs.

We tested this method by replacing of a fragment from pLysS plasmid with two fragments at the same time (Fig. 2A,B). A 255 bp of Sso7d DNA fragment and a 1007 bp of T7C565-883 DNA fragment was PCR amplified and purified. The two DNA fragments were subsequently mixed with pLysS plasmid for IOEP. A specific target overlap extension product was successfully amplified, though a non-specific product was amplified (Fig. 2C). Following PCR product purification, T4 DNA polymerase treatment and transformation, we randomly picked 8 colonies for colony PCR identification. We found that five out of the eight randomly selected colonies unambiguously contained the target gene fragment (Fig. 2D), indicating that our method can be used to efficiently clone two DNA fragments at the same time.

**Figure 3:**
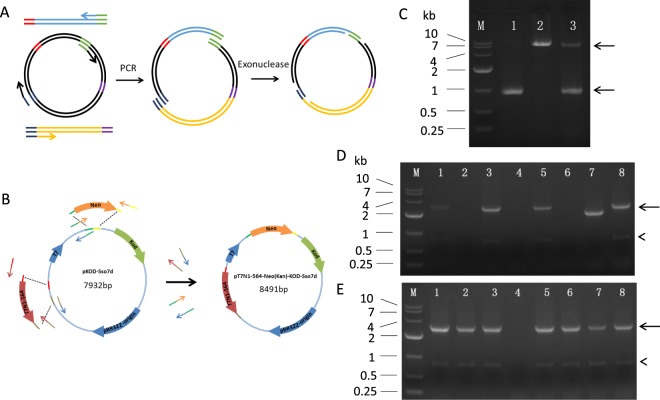
Use of IOEP for simultaneous insertion and replacement of multiple large fragments at different sites. (**A**) Scheme of the simultaneous insertion and substitution of large fragments at different sites. (**B**) Diagram for construction of pT7N1-564-Neo(Kan)-KOD-Sso7d plasmid. (**C**) Agarose gel analysis of multiple insertion and replacement PCR products. Lane 1 product was amplified by primers KanRR and T7PP, and Lane 2 product was amplified by primers KKF and ProInS. All the four primers was used for amplification Lane 3 product, which represents the single tube reaction of IOEP for multiple insertion and replacement. Please see Table 1 for primer information. The single tube (**D**) or separate tubes (**E**) reaction of IOEP, results in 4/8 and 7/8 positive colonies, respectively. Arrows indicated specific amplification. Arrowheads indicated intrinsic non-specific amplification.

We further examined whether IOEP was able to insert and replace multiple DNA fragments at different sites simultaneously (Fig. 3A,B). We amplified and purified an 866 bp of Neo DNA fragment and a 1759 bp of T7N1-564 DNA fragment by PCR. Then the Neo and T7N1-564 DNA fragments were mixed with pKOD-Sso7d plasmid for IOEP. PCR amplification was performed using two pairs of primers (KanRR and T7PP, KKF and ProInS) (Table 1) in separate tubes or in a single tube (Fig. 3C). The results showed that four out of the eight random picked colonies from the single tube reaction contain correct inserts (Fig. 3D), indicating that the single tube reaction can be used for efficient insertion of multiple fragments at different sites simultaneously. The separate tubes further increased the positive rate to seven out of eight random picked colonies (Fig. 3D).

**Table 1:** PCR Primers used in this study.

Primers	Sequences (5′-3′)
**Comparison with overlap extension PCR**	
pBS-eGFPf	CGAGGTCGACGGTATCGATATCATATGGTGAGCAAGGGCGAGGAG
pBS-eGFPr	CCGGGCTGCAGGAATTCGATATCACTTGTACAGCTCGTCCATGC
pBS-eGFPin	GATATCGAATTCCTGCAGCCCGGGGATCCACTAGTTCTAG
Cas9-cGFPf	GAGAAAGGTGGAGGCCAGCATGGAGAGCGACGAGAGCG
cGFP-WPREr	GTACCGGTACCGCGGCCGCGGAAGAGGCCGCAGAG
LCas9R-cGFP	GCTGGCCTCCACCTTTCTCTTCTTC
**Simultaneously insert two fragments into single site**	
S7dF	GCTAACGCAGTCAGGCACCAAGGAGAGATCTATGGGCGGCCGCGCAACCGTAAAGTTCA
S7dR	CCTCCAGATCCACCACCGGATCCCTTTTTCTGCTTCTCCAGCA
T7Cf	GGATCCGGTGGTGGATCTGGAGGTTCAGAAACCGTTCAGGACATCTACG
T7cDrdR	AATTCGACCCGGTCGTCGACTTACGCGAACGCGAAGTCCGA
**Simultaneously insert two DNA fragments into different sites**	
KanRF	GATAACAATTCCCCTCTAGAAGAAGGAGATATACCATGGGATCGGCCATTGAAC
KanRR	GTTAAACAAAATTATTTCTAGTCAGGTACCGAAGAACTCGTCAAGAAGG
T7NF	CCATGGGGTACTAGAGAAAGAGGAGAAAGATCTATGAACACGATTAACATCGCTAAG
T7NR	CAATGCCAGCGCTTCGTTAGTCGACGTATCCGCGGCCGCTTGAACCTCCAGATCCACCACTAGGAAGCAAGTTAAC
T7PP	GTTGGAACCTCTTACGTGCCGATCTCGATCCCGCGAAATTAATACGACTCACTATAG
KKF	CCTGACTAGAAATAATTTTGTTTAACTTTAAGAAGGAGATATACATATGATCC
ProInS	GGCACGTAAGAGGTTCCAACTTTCACCATAATGAAACACCATGGGGTACTAGAGAAAG

## Discussions

We have developed IOEP, a novel method that combines overlap extension PCR and ligation independent cloning, for efficient gene cloning and multiple site-directed large fragments insertion, deletion and replacement. Traditional OEP only uses PCR products as megaprimers, which limits its amplification efficiency with a maximum insert length of 6.7 kb^7^. As megaprimers were used only in the initial overlap extension cycles, the overlap extension product could be used by short primers for exponential amplification, IOEP can be used to efficiently amplify a 12 kb difficult–to-amplify overlap extension product, which is a challenge for OEP (Fig. 1B,C). The results suggested that the size limit of IOEP should be mainly depended on the DNA polymerase’s intrinsic property in PCR to amplify large fragment, as different DNA polymerase varied greatly in amplification of large and difficult-to-amplify product^19^. Benefited from the exponential amplification, only a tiny amount of plasmid DNA was used during IOEP, thus makes the treatment of the amplified product by DpnI to remove original plasmid an optional procedure.

To our knowledge, insertion of multiple fragments larger than hundreds of basepairs at multiple sites simultaneously has not been reported previously. Multiple site-directed mutagenesis, a widely used method which utilized oligonucleotides for deletion, insertion and substitution of multiple sites in plasmid, can be used to delete large fragment in plasmid, but the inserted fragment are limited to tens of nucleotides^17,20^. Exonuclease-based methods can be used to improve the mutation efficiency of multiple site-directed mutagenesis^17^, they were only used for multiple large fragments insertion at a single site of a plasmid^14–18^. Here, we demonstrated that IOEP can be used to insert or substitute multiple large fragments simultaneously at multiple sites (Fig. 3), indicating a broader application of this method.

## Methods

### Bacterial strains, plasmids, and media

Competent *Escherichia coli* DH5α (Transgen Biotech) was used as the host for the DNA assembly. Plasmid pCDH-CMV-MCS-EF1-copGFP (System Biosciences) was used as the template to amplify the copGFP-WPRE DNA fragment. The plasmid LCas9 was used as the vector for the assembly of copGFP-WPRE DNA fragments. The Rosetta (DE3)(CWBiotech) was used as the template to amplify the T7N1-564 and T7C565-883 DNA fragments. Plasmid pCR2.1 (ThermoFisher Scientific) was used as the template to amplify the Neo DNA fragment. Plasmid pKOD-Sso7d was used as the template to amplify the Sso7d DNA fragment also as the vector for assembly of Neo and T7N1-564 DNA fragments. The plasmid pLysS (Novagen) was used as the vector for the assembly of Sso7d and T7C565-883 DNA fragments. LB broth (10 g/L tryptone, 5 g/L yeast extract, 10/g L NaCl) was used for cloning and cell culture, supplemented with ampicillin (100 μg/mL) or chloramphenicol (34 μg/mL) when necessary.

### PCR conditions

All PCRs were carried out in 200 μl thin-walled PCR tubes using 25 μl of reaction mixture, multiple tubes were used for scaling up the amplification if necessary. All the primers (please see Table 1 for sequence information) were synthesized by Sangon Biotech (Shanghai, China), and used at a final concentration of 0.4 μM if not specified. PrimeSTAR GXL DNA polymerase (Takara Biotech Co. Ltd., Dalian, China) was used at 0.625 U per reaction. All PCRs were performed under the following programs: 98 ^o^C pre-denaturation for 30 s; followed by 30 cycles of denaturation at 98 ^o^C for 10 s, annealing at 60 ^o^C for 30 s, and extension at 68 ^o^C for 60 s (varied at approximately 30 s/kb DNA fragment); and a final extension at 68 ^o^C for 5 min.

### Comparison with overlap extension PCR

To compare the efficiency of OEP and IOEP, we firstly checked the GFP incorporation into pBlueScript II KS (−). First round PCR: 1 ng of pET-EGFP plasmid DNA was used as the template, pBS-eGFPf and pBS-eGFPr (Table 1) were used as primers. The PCR extension time was 30 s. The PCR product was purified by DNA Clean-up Kit (CWBIO). Purification the first round PCR product by agarose gel extraction is optional in most cases, but is necessary if there is substantial non-specific amplification. Second round PCR: 50 ng of the GFP DNA fragment was mixed with 100 ng of pBlueScript II KS (−) plasmid DNA for OEP, and the amount of GFP DNA fragment and pBlueScript II KS (−) plasmid DNA was decreased to 10 ng and 1 ng, respectively for IOEP. A final concentration 0.4 μM of pBS-eGFPr and pBS-eGFPin were used as primers for IOEP. The PCR extension time was 2 min. The PCR products were purified by DNA Clean-up Kit (CWBIO). Prior transformation, 20 ng of the OEP product was mixed with 1 μl of FastDigest DpnI (ThermoFisher Scientific) to digest the template DNA, whereas 20 ng of the IOEP products was treated with or without (IOEP-) T4 DNA polymerase.

We further tested the efficiency of IOEP for a more difficult plasmid construction. First round PCR: 1 ng of pCDH-CMV-MCS-EF1-copGFP plasmid DNA was used as the template, Cas9-cGFPf and cGFP-WPREr (Table 1) were used as primers. The PCR extension time was 1 min. The PCR product was purified by DNA Clean-up Kit (CWBIO). Second round PCR: 50 ng of the copGFP-WPRE DNA fragment was mixed with 100 ng of LCas9 plasmid DNA for OEP, and the amount of copGFP-WPRE fragment and LCas9 plasmid DNA was decreased to 10 ng and 1 ng, respectively for IOEP. A final concentration 0.4 μM of Cas9-cGFPf and LCas9R-cGFP were used as primers for IOEP. The PCR extension time was 5 min. After purification by DNA Clean-up Kit (CWBIO), the second round PCR product of OEP was mixed with 1 μl of FastDigest DpnI (ThermoFisher Scientific) to digest the template DNA, whereas the second round PCR product of IOEP was treated by T4 DNA polymerase.

### Simultaneously insert two fragments into single site

First round PCR: 1 ng of KOD-S7 plasmid and Rosetta (DE3) genomic DNA were used as template, and S7dF & S7dR, T7cF & T7cDrdR were used as primer pairs for amplification of the Sso7d DNA fragment and T7C565-883 DNA fragment, respectively. The PCR extension time was 30 s. The PCR products were purified by DNA Clean-up Kit (CWBIO). Second round PCR: 1 ng of both the purified Sso7d DNA fragment and T7C565-883 DNA fragment were mixed with 1 ng of pLysS, and 0.4 μM of primers S7dR and T7cF were used. The PCR extension time was 2 min.

### Simultaneously insert two DNA fragments into different sites

First round PCR: 1 ng of pCR2.1 DNA was used as the template, and the primers KanRF and KanRR were used to amplify Neo DNA. One nanogram of Rosetta (DE3) DNA was used as the template, and three primers T7NF, T7NR and ProInS were used for T7N1-564 DNA fragment amplification. A final concentration of 0.1 μM of T7NF, and 0.4 μM of T7NR and ProInS were used. To avoid too long and too expensive of the T7NF to be synthesized, the third primer ProIns, whose 3′ end is overlapping with the 5′ end of T7NF, was used to introduce a bacterial promoter into the T7N1-564 DNA fragment. The PCR extension time was 1 min. Second round PCR: 10 ng of Neo DNA fragment, 10 ng of T7N1-564 DNA fragment and 1 ng of pKOD-Sso7d plasmid DNA were mixed with two pairs of primers (KanRR and T7PP, KKF and ProInS) together (single tube reaction) or with one pair per tube (separate tube reactions). The PCR extension time was 5 min.

### T4 DNA polymerase treatment and transformation

T4 DNA polymerase, which has strong 3′-5′ exonuclease, was as an exonuclease to generate complementary single-stranded DNA ends for IOEP to enhance its transformation efficiency. Briefly, in a 10 μL reaction, a total amount of 100 ng DNA fragment(s) obtained by IOEP was mixed in equal molar ratio in 1X T4 DNA polymerase buffer (Sangon) or 1X CutSmart buffer (NEB), 1 U of T4 DNA polymerase (Sangon) was added before incubating at 20 °C for 2 min to recess the DNA for generating of single-stranded regions at the DNA ends, followed by heating to 75 °C for 5 min to inactivate T4 DNA polymerase, then the reaction was cooled to 50 °C for 10 min to allow the annealing of complementary DNA ends^16,17^. An aliquot (5 μl) of each T4 DNA polymerase treated product was used to transform 50 μl of commercial chemically competent *E*. *coli* DH5α cells (Transgen Biotech) using standard protocol.
